# Echocardiographic Assessment of Cardiac Structural and Functional Indices in Broiler Chickens Treated with Silver Nanoparticles

**DOI:** 10.1155/2013/931432

**Published:** 2013-10-27

**Authors:** Hamid Raieszadeh, Vahid Noaman, Mehrdad Yadegari

**Affiliations:** ^1^Postgraduate Veterinary Medicine, Shahrekord Branch, Islamic Azad University, Shahrekord, Iran; ^2^Department of Veterinary Research, Isfahan Research Center for Agriculture and Natural Resources, P.O. Box 81785-199, Isfahan, Iran; ^3^Department of Radiology, Faculty of Veterinary Medicine, Shahrekord Branch, Islamic Azad University, Shahrekord, Iran

## Abstract

270 Ross broiler chickens of twenty days old were housed in 18-floor pens in a completely randomized design with six treatment groups and three replicate groups and fifteen chicks per each pen. The treatment groups (1–6) consisted of 0, 10, 20, 30, 50, and 70 ppm of nanocide in drinking water, respectively. At 26 days of age, 3 chickens were selected randomly for echocardiography using a 7.5 MHz linear probe, and the left ventricular internal diameter at the end of diastole (LVIDd), left ventricular internal diameter at the end of systole (LVIDs), left ventricular fractional shortening (LVFS), ejection fraction (EF), stroke volume (SV), interventricular septum thickness at the end of systole (IVSTs), and interventricular septum thickness at the end of diastole (IVSTd) were evaluated. LVIDd and LVIDs in group six were of higher rate than other groups and showed statistically significant differences with groups two, three, and four (*P* < 0.05). LVFS, percentage of EF, and IVSTd were minimum in group six and had significant difference with other groups (*P* < 0.05). The results of this study showed that prescription of high dosage of nanocide leads to cardiovascular problems with decrease in myocardial contractility and increase in the internal diameter of left ventricle.

## 1. Introduction

 Silver is a noble metal that has been known since ancient time to control microbial proliferation even against antibiotic-resistant bacteria [1–3]. After clarifying the properties of silver in burn wound healing and its antihemorrhagic and anti-inflammatory effect, silver was used more than previously [4–8]. Recent studies on use of silver in nanosize as an alternative to antibiotics and its preprobiotic properties with increasing immunity have led to use of this nanoparticle largely, especially in veterinary and dependent sciences [9–12]. 

Not only widespread use of nanosilver in low levels of silver ions promotes rapid development of bacterial resistance but also use of nanosilver in high levels of silver ions causes toxicity in human and animals [13]. *In vitro *studies have found that nanosilver was toxic to mammalian liver cells, stem cells, and even brain cells [14–16].

Early examples in agriculture include the use of nanosilver as a “nanobiotic” in poultry production [17], and in recent years, the quantities of nanosilver consumed in broiler chicken farms of Iran as nano-biotic have increased considerably. Despite continued and the widespread use of nanosilver in poultry farms, to our knowledge, there is very little information about the effects of nanosilver on the vital organs, especially cardiac structure and cardiac function of chickens. Various aspects of echocardiography (such as one-dimensional method, two-dimensional method and doppler method) are used for definiting anatomy of heart, evaluating cardiac structure and function, and measuring the heart cavities and coronary blood flow patterns in different species of human, horse, dog, and cat [18, 19]. As regarded ultrasound is valuable, and noninvasive diagnostic applications in veterinary medicine and in many cases can be replaced by other imaging methods to assess cardiac structure and function [20–22]; this method was used in this study. Therefore, the present study was conducted to evaluate the effect of different doses of nanosilver on cardiac structure and function indicators by echocardiographic technique. 

## 2. Materials and Methods

**Figure 1:**
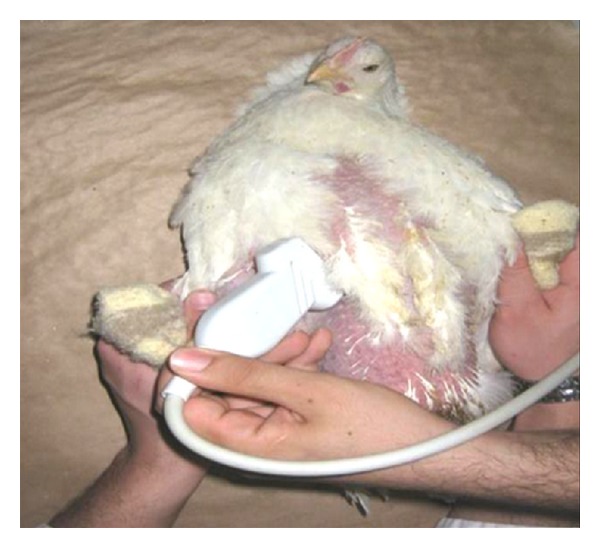
Position of transducer which is used for imaging echocardiography in chicken.

**Figure 2:**
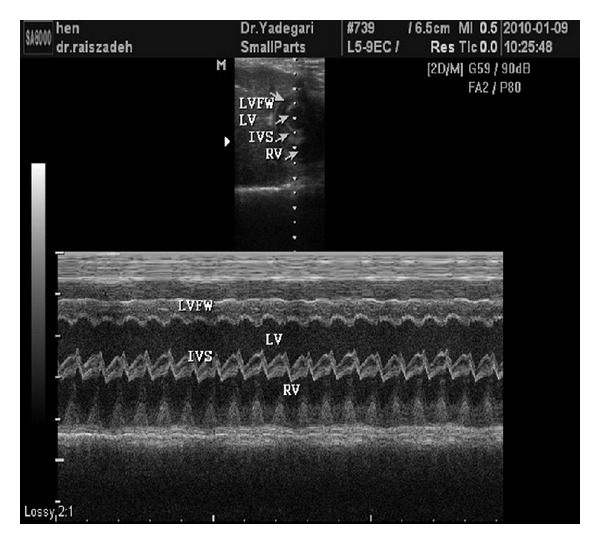
B-mode and M-mode echocardiography. Left ventricle (LV), right ventricle (RV), left ventricular free wall (LVFW), and wall between the two ventricles (IVS).

The study was carried out on 270 Ross broiler chickens twenty days old with about the same weight. The Chicks were of housed in 18-floor pens in a completely randomized design with six treatment and three replicate groups and fifteen chicks per each pen. The birds received water and food *ad libitum*, in feeders and linear troughs. The treatment groups consisted of different concentrations of nanocide in drinking water (group 1: 0 ppm, group 2: 10 ppm, group 3: 20 ppm, group 4: 30 ppm, group 5: 50 ppm, and group 6: 70 ppm). The active ingredient of this combination is colloidal silver with nanoside brand which is made in Behban chemical Co. At 26 days of age from each pen (repeat), 3 chickens were selected randomly and were transferred to the examination hall. After stress reduction and calming the bird, echocardiography was performed from each chick. The sternal feathers (echocardiography probe placement) slowly and without bleeding were short, and chickens for echocardiographic studies were transferred to hospital ultrasound room. With appropriate adjustments in the light, chicks were in the lowest stress. Chicken echocardiograms have been taken using a 7.5 MHz linear probe. Chickens were in dorsal recumbency. The most common approach is ventromedial, just caudal to the sternum, viewing the heart through the liver. Patients may be held in partial upright dorsal recumbency or completely vertical position for this approach [23]. In this study, given that the left chambers of heart are examined, All the best images are obtained when the bird is in the dorsal recumbency and probe is placed in the right parasternal just in front of the stifle joint (Figure 1). In this study, echocardiography was performed in two methods B-mode and M-mode. For overall and large assessment of the heart two-dimensional echocardiographic method or B-mode and to accurately measure cavity and distance between them, the M-mode echocardiography was used. At first, heart was investigated by the presence or absence of pericardial effusion. Distance between two troughs and distance between two peaks was used for measurement of left ventricular internal diameter in the end of diastole (LVIDd) and left ventricular internal diameter in the end of systole (LVIDs), respectively (Figure 2). Interventricular septum thickness in the end of diastole (IVSTd) and inter ventricular septum thickness in the end of systole (IVSTs) were measured for each sample separately. Fractional shortening (FS) or “end-diastolic diameter minus end-systolic diameter divided by end-diastolic diameter” was calculated for left ventricle (LVFS) [24, 25]. Stroke volume (SV) or “left ventricular diastolic volume (LVVd) minus left ventricular systolic volume (LVVs)” and ejection fraction (EF) or “stroke volume divided by LVVd” were evaluated [26, 27]. The data were analyzed by the GLM procedure of SAS software. Mean values, standard deviations, and statistical differences were calculated among treatment groups. Duncan's multiple range tests were used to determine differences among the means. Statistically significant level of difference was considered at *P* < 0.05. 

## 3. Results

**Figure 3:**
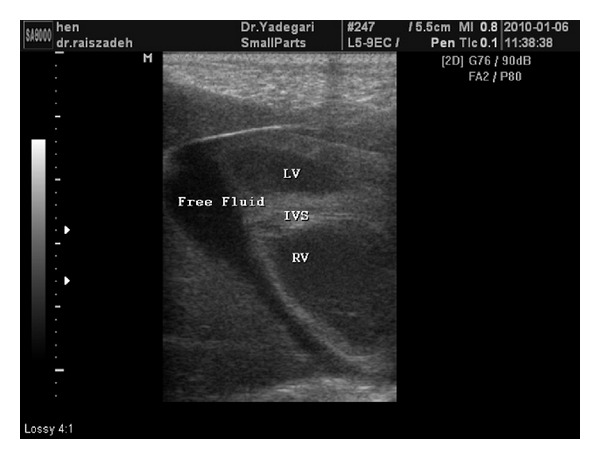
Pericardial effusion in group six.

With clear M- and B-mode images, all data were collected. LVIDd and LVIDs in group six were of higher rate than other groups and showed statistically significant differences with groups two, three, and four (*P* < 0.05). LVFS was minimum in group six and had significant difference with groups one, two, and three (*P* < 0.05). Percentage of EF was minimum in group six and had significant difference with groups two and three (*P* < 0.05). IVSTd was minimum in group six and had significant difference with groups one, four, and five (*P* < 0.05). No significant difference was observed in IVSTs in different groups. Stroke volume in groups five and six was slightly higher than other groups, but those differences were not statistically significant (Table 1). In B-mode, echocardiographic images clearly observed pericardial effusion in groups five and six (Figure 3). 

## 4. Discussion

Antibacterial and antiseptic effects of silver ion in preventing infection with coli forms, staphylococcus, and streptococcus have been proven in previous studies [26–29]. Silver nanoparticles can destroy many kinds of bacteria, viruses, and fungi, and it is recommended that it can be used in the treatment of certain viral diseases in animals like Influenza and Newcastle [30]. However, extended and excessive exposure to the silver nanoparticles, like many other drugs, has side effects and pathologic effect on living tissues. Sung et al. and other researchers indicated that the use of nanoscale silver in the drinking water and inhalation has toxic effects (hyperplasia, fibrosis, and vacuolation) on organs such as the liver and lungs [31, 32]. Heart as a vital organ of the birds is sensitive to different medications and shows different reactions [33]. Despite the widespread use of nanosilver products in poultry farms, relatively few studies have been undertaken to determine the biological effects of nanosilver exposure [34]. Because of the lack of information on the effects of silver nanoparticles on the heart, in this study, the effects of different doses of nanosilver on cardiac structure and function were evaluated. 

Absorbed nanosilvers bind to plasma proteins and can enter the cells. They are distributed in organs such as liver, kidney, heart, lymph nodes, brain, lung, stomach, and testicles [35]. The rate of silver retention is not the same in different organs; this situation may be related to activity, blood flow rate in the organs [36]. Recently, it was reported that nanoparticles and nanomaterials generate free radicals and oxidative stress [16]. The results of research showed that silver nanoparticles can damage different organs and tissues [15]. Cytotoxicity of silver nanoparticles to the mitochondrial activity increased with the increase in the concentration of silver nanoparticles, and silver nano-particles are the most toxic with drastic reduction of mitochondrial function, increased membrane leakage, necrosis, and induction of apoptosis [34]. Cardiac necroses and decrease of blood glutathione per oxidase have been reported in silver treated swine [37]. LVFS is the most important indicator of heart function, and in the present study, in group six (70 ppm), decreased LVFS was observed. Indeed, increasing nanosilver consumption causes heart and lung damage [34]. Reduced lung capacity and myocardial lesions cause pulmonary hypertension, and ascites also contribute to the development of compensatory right ventricular hypertrophy [38], dilated myopathy, and decreased LVFS. In dilated myopathy, the heart size is increased without increasing the thickness of the heart muscle, even the walls are thinner to increase the size of chambers [33]. Following this, the increase will occur in LVIDd and LVIDs.

According to formula FS% = (LVIDd − LVIDs) × 100/LVIDd and the direct connection between LVIDd and LVFS, increased percentage of LVFS in groups that developed this complication is probably due to the above changes. Deng et al. in a study on heart parameters of chickens with ascites syndrome and normal chickens showed an increase in LVDs and a decrease in FS of chickens with ascites syndrome [39]. In another study, cardiac echocardiography on chickens with pulmonary hypertension syndrome (PHS) showed that FS in right and left ventricles was decreased and RVID in the end of systole and diastole was increased [40].

Stroke volume is the volume of blood ejected per heart beat and influenced by preload, afterload, and contractility [24]. Reduction of inter ventricular thicknesses and heart muscle myopathy in groups with high dose of nano silver results in myocardial contractility reduction which consequently led to the stroke volume reduction.

According to ejection fraction formula EF% = (SV × 100/LVVd) with decreasing stroke volume, the percentage of ejection fraction in groups with high doses of nanosilver especially group six is reduced.

Pericardial effusion is an abnormal accumulation of fluid in the pericardial cavity, that is, a pathological condition [41]. In this study, pericardial effusion in the group with high dose of nanosilver probably was the result of silver toxicity and consequently myocardial and liver damage that leads to blood reject from the heart, passive congestion, and consequently hydropericardium and ascites. 

## 5. Conclusion

Recent study showed negative effects of high dose silver nano particle on cardiac structure and function in chicken. In addition,the survey results showed that the most important changes in cardiac parameters can be due to decreased cardiac contractility that probably has occurred due to toxicity with high dose of nano silver.

## Figures and Tables

**Table 1:** Comparison of echocardiographic indices (Means ± Standard deviation) among different chicken groups treated with nanoparticles.

Treatment groups	Cardiac parameters^1^						
	LVIDd	LVIDs	LVFS	EF	IVSTd	IVSTs	SV
1 (0 ppm)	1.08 ± 0.03^ab^	0.56 ± 0.02^ab^	47.82 ± 0.92^a^	83.77 ± 0.98^ab^	0.34 ± 0.02^a^	0.42 ± 0.02	2.19 ± 0.11
2 (10 ppm)	1.04 ± 0.02^b^	0.51 ± 0.02^b^	51.29 ± 1.30^a^	85.36 ± 1.62^a ^	0.32 ± 0.01^ab^	0.43 ± 0.03	2.02 ± 0.15
3 (20 ppm)	1.02 ± 0.01^b^	0.50 ± 0.03^b^	50.77 ± 3.57^a^	85.48 ± 2.41^a^	0.32 ± 0.01^ab^	0.43 ± 0.01	2.09 ± 0.30
4 (30 ppm)	1.01 ± 0.02^b^	0.53 ± 0.01^b^	47.70 ± 0.52^ab^	83.05 ± 0.54^ab^	0.36 ± 0.01^a ^	0.40 ± 0.03	1.88 ± 0.09
5 (50 ppm)	1.03 ± 0.02^ab^	0.55 ± 0.06^ab^	46.42 ± 5.75^ab^	81.61 ± 5.15^ab^	0.33 ± 0.03^a^	0.36 ± 0.04	1.80 ± 0.16
6 (70 ppm)	1.09 ± 0.06^a^	0.62 ± 0.05^a^	42.32 ± 1.82^b^	79.40 ± 3.95^b^	0.29 ± 0.01^b^	0.38 ± 0.01	1.84 ± 0.07

^a-b^Different superscript letters in each column indicate significant difference between groups (*P* < 0.05).

Data are reported as mean ± SD.

^1^LVIDd: left ventricular internal diameter in the end of diastole; LVIDs: left ventricular internal diameter in the end of systole; LVFS: left ventricular fractional shortening; EF: ejection fraction; IVSTd: interventricular septum thickness in the end of diastole; IVSTs: inter ventricular septum thickness in the end of systole; SV: stroke volume.
